# Two Web-Based and Theory-Based Interventions With and Without Brief Motivational Interviewing in the Promotion of Human Papillomavirus Vaccination Among Chinese Men Who Have Sex With Men: Randomized Controlled Trial

**DOI:** 10.2196/21465

**Published:** 2021-02-02

**Authors:** Zixin Wang, Joseph T F Lau, Tsun Kwan Mary Ip, Yebo Yu, Francois Fong, Yuan Fang, Phoenix K H Mo

**Affiliations:** 1 JC School of Public Health and Primary Care Faculty of Medicine The Chinese University of Hong Kong Hong Kong China; 2 NeoHealth Hong Kong China; 3 Department of Early Childhood Education Faculty of Education and Human Development The Education University of Hong Kong Hong Kong China

**Keywords:** HPV vaccination, web-based health promotion, randomized controlled trial, men who have sex with men, China, mobile phone

## Abstract

**Background:**

Human papillomavirus (HPV) vaccination is effective in the prevention of vaccine-type genital warts and cancers among men who have sex with men (MSM).

**Objective:**

The primary objective of this randomized controlled trial (RCT) is to evaluate the efficacies of 2 web- and theory–based interventions with and without brief motivational interviewing (MI) over the phone to increase the completion of HPV vaccination among unvaccinated participants within a 24-month follow-up period compared with the control group.

**Methods:**

A 3-arm parallel-group RCT was conducted between July 2017 and December 2019. Five telephone surveys were conducted at baseline and at 3, 6, 9, and 24 months by blinded interviewers. Participants were Hong Kong Chinese–speaking MSM aged between 18 and 45 years with regular internet access who were recruited from outreaching at venues, web-based recruitment, and peer referral. Those who had ever received HPV vaccination were excluded. A total of 624 participants were randomized into either the online tutorial (OT) only group (n=208), the OT plus MI group (OT-MI; n=208), or the control group (n=208). In total, 459 (459/624, 73.6%) completed the follow-up evaluation at 24 months. Participants in the OT group received a fully automated OT developed based on the health belief model. On top of the same OT, the OT-MI group received brief MI over the phone. Reminders were sent to the participants of the OT and OT-MI groups after 1, 2, 4, 6, and 8 months. Participants in the control group received web-based health communication messages unrelated to HPV or HPV vaccination. The research team validated the self-reported HPV vaccination uptake. Intention-to-treat analysis was used for outcome analyses. Logistic regression models and multivariable linear regression models were used to test the between-group differences in primary and secondary outcomes. Baron and Kenny’s methods were used to test the mediation hypothesis.

**Results:**

The participants in the OT-MI group reported a significantly higher validated completion of HPV vaccination at 24 months than the control group (36/208, 17.3% vs 15/208, 7.2%; *P*=.006). However, the difference in HPV vaccination completion between the OT and the control groups (24/208, 11.5% vs 15/208, 7.2%; *P*=.17), or between OT-MI and OT groups (*P*=.13), was not statistically significant. The association between randomization status (OT-MI group vs control group) and HPV vaccination completion became statistically nonsignificant after controlling for changes in the perceived susceptibility to HPV (24 months vs baseline), whereas perceived susceptibility remained strongly associated with HPV vaccination uptake in the model (*P*<.001). Changes in perceived susceptibility fully mediated the intervention effect.

**Conclusions:**

Theory-based OT with brief MI over the phone was effective in increasing HPV vaccination completion among Chinese MSM. Perceived susceptibility is an active theoretical component that causes behavioral changes.

**Trial Registration:**

ClinicalTrials.gov NCT03286907; https://clinicaltrials.gov/ct2/show/NCT03286907

## Introduction

Men who have sex with men (MSM) are at a high risk of contracting human papillomavirus (HPV) and its related diseases (eg, genital warts and penile or anal cancers) [1]. Meta-analyses have reported that the overall prevalence of genital HPV infection was very high among MSM both internationally (63.9% in HIV-negative MSM and 92.6% in HIV-infected MSM) [2] and in China (66.3% among MSM in general) [3]. This prevalence was much higher than in MSM than that in the general male population (eg, 12.4% in Europe and 16.9% in China) [4-6]. In addition to the high prevalence of genital warts [7], the anal cancer risk of MSM is 32 to 52 times higher than that in the general population [8]. The HPV-related cancer risk was the highest among HIV-infected MSM, which accounted for 9.9% of the MSM population in China in 2016 [9]. Although there is a lack of data about the prevalence or incidence of HPV-related diseases among MSM in Hong Kong, such prevalence may be much higher among MSM than that among the general male population (genital warts prevalence 0.94% and incidence 292.2 per 100,000 person-years) [10].

HPV vaccination is highly effective in the prevention of vaccine-type genital warts and cancers among MSM [11,12]. A study also showed that people who are already infected with one or more HPV types can still receive protection from other HPV types with the vaccines [13]. In addition, HPV vaccination could also prevent the development or recurrence of subsequent HPV-related diseases (eg, anal intraepithelial neoplasia) among people with a history of HPV infection [14-16]. Modeling work found that HPV vaccination for MSM could have a substantial impact on HPV-related disease incidence in this population and is cost-effective [17]. As a result, the US Centers for Disease Control and Prevention (CDC) recommends MSM who are in the age range of 45 years or below to receive HPV vaccination [13,18]. Free national HPV vaccination programs for MSM aged up to 45 years attending sexual health and HIV clinics rolled out in England and Scotland in 2018 and 2017, respectively [19,20]. The Victorian Government of Australia funded a free time-limited HPV vaccination catch-up program for MSM aged up to 26 years in 2017 [21]. The recommended course for MSM is 3 doses administered within 1 year; however, a 24-month period is clinically acceptable (second and third doses administered at least one month and 3 months after the first and second doses, respectively) [22]. However, in Hong Kong, no free or subsidized HPV vaccination program targeted MSM or other male populations. Two types of HPV vaccines (4-valent and 9-valent) are provided to males aged 9 years or more at the cost of HK $2500 to HK $4000 (US $321 to US $514) for a 3-dose course by private physicians in Hong Kong [23].

The uptake of free HPV vaccination provided by national programs was 37.6% among MSM in the United States [24], 42.6% in Australia [21], 49.1% in England [25], and 63.7% in Scotland [20]. HPV vaccination uptake among the male population is very low in Hong Kong [26], and previous studies have reported zero uptake among MSM [27,28]. About 30% of MSM in Hong Kong intended to take up HPV vaccination at the market rate [27,28]. Our literature search identified 4 studies that promoted HPV vaccination among MSM [29-32]. In a pilot intervention study conducted in the United States, young MSM were recruited via a popular web-based dating app and linked to a mobile health (mHealth) tool providing information and fostering access to HPV vaccination [31]. Of the 42 MSM who engaged with the mHealth tool, 11 (26%) received HPV vaccination [31]. Another web-based intervention was developed to provide information related to HPV prevalence among MSM in the United States, the effectiveness of the HPV vaccination, how to address potential barriers of receiving HPV vaccination, vaccine costs, and health insurance. A pilot randomized controlled trial (RCT) showed that such intervention was effective in increasing self-reported HPV vaccination initiation (receipt of 1 or more doses) compared with providing simple information about the vaccines (34/76, 45% vs 19/74, 26%; *P*=.02). However, the intervention did not increase HPV vaccination completion (receipt of 3 required doses: 8/76, 11% vs 2/74, 3%; *P*=.07). An ongoing RCT is comparing the efficacy of the same web-based intervention plus 2 different types of reminders (interactive vs noninteractive) versus the provision of simple HPV vaccination information among MSM in the United States [30]. In addition, an RCT tested the efficacy of texting-based intervention in the promotion of HPV vaccination among young MSM in the United States [32]. Participants who were assigned to the intervention group received daily SMS text messages for the first 3 weeks related to HPV vaccination, whereas those in the control group received SMS text messages related to other sexual health topics [32]. The intervention was effective in promoting HPV vaccination initiation (19.4% vs 6.6%) [32]. To our knowledge, no HPV vaccination promotion targeting MSM was conducted outside the United States.

A meta-analysis showed that theory-based interventions were more effective than nontheory-based interventions [33]. Our formative studies showed that some constructs of the health belief model (HBM) were significantly associated with the willingness to take up HPV vaccination at the market rate among local MSM [27,28]. These constructs included perceived susceptibility (risk of HPV, genital warts, and penile or anal cancer), perceived severity (chance of HPV infection causing genital warts and penile or anal cancers), perceived benefit of HPV vaccination (efficacy in preventing genital warts and penile or anal cancers), perceived barriers (high cost, potential side effects, and embarrassment), and cue to action (recommended by doctor or peers to take up the vaccines). Such findings were considered while developing the web-based interventions used in this study. Video was produced to promote HPV vaccination, as the audio-visual approach has been used effectively in health promotion programs. The authors also conducted brief motivational interviewing (MI) over the phone to promote in one of the intervention groups. MI is a client-centered, nondirective, goal-oriented counseling technique, which helps clients explore and resolve any ambivalence that might have to change [34]. The US CDC lists MI among its best evidence intervention [35]. A systematic review indicated that MI was acceptable to MSM and feasible to deliver over the phone [36]. A telephonic delivery of MI is useful in changing HIV or sexually transmitted infection (STI)–related behaviors among MSM [37].

The primary objective of the RCT is to evaluate the relative efficacies of 2 theory- and web-based interventions with and without brief MI over the phone in increasing the completion of HPV vaccination (receipt of 3 required doses) within a 24-month follow-up period among unvaccinated Hong Kong Chinese MSM compared with the control group. The secondary objective is to evaluate the relative efficacies of the interventions in changing constructs related to the HBM over the follow-up period.

## Methods

### Study Design

**Figure 1:**
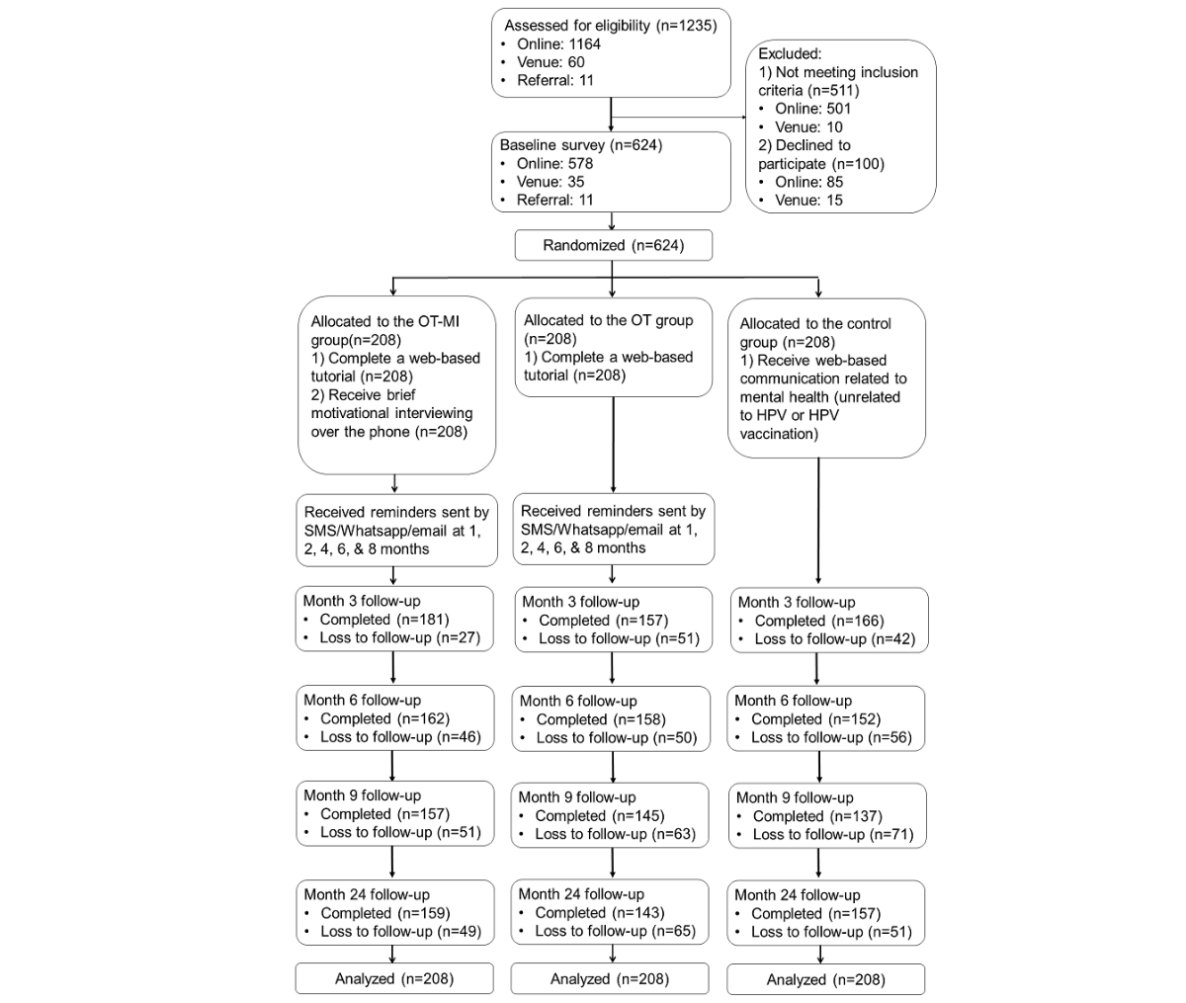
CONSORT (Consolidated Standards of Reporting Trials) flow diagram of the study.

A 3-arm parallel RCT was conducted between July 2017 and December 2019. This study was registered at ClinicalTrial.gov (number NCT03286907). The consort flowchart is shown in Figure 1.

### Participants

Participants were (1) Hong Kong Chinese–speaking men aged between 18 and 45 years; (2) self-reported oral or anal intercourse with at least one man in the past 6 months; (3) having regular internet access; and (4) willing to complete telephonic follow-up evaluations. Those who had ever received HPV vaccination were excluded. We also excluded MSM aged over 45 years because HPV vaccination was not approved for use in this age group [18].

### Recruitment Procedures

Participants were recruited through multiple sources. Recently, the Hong Kong government geographically located all venues frequently visited by local MSM, including 12 gay bars and 16 gay saunas [38]. Upon approval of the owners, trained and experienced fieldworkers approached prospective MSM participants in these venues at different time slots during weekdays and weekends. These fieldworkers briefed prospective participants about the study details and gave them an information sheet. The research team also conducted a web-based outreach by periodically posting information about the study as discussion topics on 2 gay websites with the highest traffic in Hong Kong. If prospective participants were interested in this study, they could contact the interviewers through private messaging or other means (eg, WhatsApp, telephone, and email). Recruitment was supplemented by peer referrals. Participants were guaranteed anonymity during the study and had the right to discontinue participation in the study at any time. Their refusal or withdrawal from the study would not affect their access to any future services. Verbal instead of written informed consent was obtained so as to maintaining anonymity, but the fieldworkers signed a form pledging that the participants had been fully informed about the study. Multiple forms of contact information were obtained to make an appointment to conduct a baseline telephonic interview. Upon appointment, trained telephone interviewers confirmed the eligibility and consent of the participants to participate in the study and conducted telephone interviews, which took about 10 min to complete. The follow-up surveys at 3, 6, and 9 months only recorded HPV vaccination uptake (number of doses, price, venue, date, and side effects). Participants who presented at 24 months were asked about HPV vaccination history and perceptions based on the HBM. Up to 5 calls were made at different timeslots during weekdays or weekends before considering a case as loss-to-follow-up. Upon completion of each of the 5 surveys, an HK $50 (US $6.3) supermarket or café coupon was mailed to participants as compensation for their time. Telephone numbers and addresses were cross-checked to avoid repetition. Ethics approval was obtained from the Survey and Behavioral Research Ethics Committee of the Chinese University of Hong Kong (ref no.: 13141651) and the Joint Chinese University of Hong Kong – New Territories East Cluster Clinical Research Ethics Committee (ref no.: 2015.687-T).

Among 1235 prospective participants who were approached, none had received HPV vaccination. A total of 724 MSM were eligible for participation after screening, 100 refused to participate in the study for time and/or other logistical reasons, and 624 completed the baseline survey. The response rate was 86.2%. The number of participants who completed the follow-up surveys was 504 (504/624, 80.8%) at 3 months, 472 (472/624, 75.6%) at 6 months, 439 (439/624, 70.4%) at 9 months, and 459 (459/624, 73.6%) at 24 months, respectively.

### Development of Intervention Materials

A panel comprising 3 MSM volunteers, 2 epidemiologists, 1 health psychologist, 1 health communication expert, 2 experienced nongovernmental organization (NGO) workers, and 1 video producer was formed. A literature review and a discussion group that involved 5 MSM were conducted to inform the design of the web-based health communication messages. The web-based tutorial video was produced by a professional team, reviewed by 3 other MSM, and finalized by the panel.

### MI Training and Ongoing Supervision

Fieldworkers received 2 full days (14 hours in total) of basic MI training by an experienced trainer who is a member of the MI Network of Trainers. In addition, fieldworkers were trained to deliver the intervention using role-plays over the phone to increase their confidence. The role-plays and protocol-specific practice were conducted twice a week for 4 weeks and maintained by coaching sessions once every 2 weeks by the same trainer. Fieldworkers were not deployed in the fieldwork unless they had achieved a beginner level of competence. In addition, fieldworkers also received a 4-hour training workshop about background knowledge of local MSM, HPV-related diseases, and HPV vaccination. Meetings for problem solving and improvements were held 2 weeks after the commencement of fieldwork and monthly afterward. Fieldworkers sought support from the trainer via phone calls during the project period.

### The Baseline Survey and Random Allocation Process

After completing the baseline telephone survey, the participants were randomized 1:1:1 to either the control group, the OT group, or the OT-MI group. Computer-generated random allocation codes were produced and sealed in opaque envelopes by a research staff member with no involvement in recruitment or baseline survey. One envelope was drawn and opened by the interviewers. They then informed the participant which group they were assigned to. Block randomization with a block size of 12 was used.

### Intervention for the OT Group

A unique login identity and a link to access a web-based self-administered tutorial were sent to participants in the OT group. Next, the participants were requested to visit this website and performed the self-administered tutorial within 3 days. Up to 5 reminders (in the one-week period) were sent to those who did not log in within 3 days via email or social media channels.

The first part of the online tutorial (OT) was a 5-min web-based video. The carefully designed contents were based on theory-based factors associated with the acceptability of HPV vaccination among local MSM [27,28] and guided by the HBM [39]. In the video, a peer MSM discussed the high risk of having penile or anal cancers among MSM, severe consequences of genital warts, penile or anal cancers, promising efficacy, and long protection duration of HPV vaccination in the prevention of genital warts and penile or anal cancers. Flashes of scary images of genital warts on penis/anal and penile and anal cancers were shown in the video to increase perceived severity. The peer MSM also emphasized that HPV vaccination is a worthy long-term investment for their health and demonstrated the procedures for receiving HPV vaccination in collaborative private clinics, which portrayed caring, guaranteed privacy, and a nonjudgmental environment. To ensure a complete exposure, the video was formatted in such a way that the participants could not fast-forward or skip any part of it.

The second part of the OT was a self-administered exercise. The participants answered some web-based multiple choice questions (eg, knowledge of HPV and HPV vaccination) and performed a short exercise to modify related cognitions (eg, “Please list three potential benefits you will gain after taking up HPV vaccination”). Model answers were displayed for incorrect entries, and the participants were requested to re-enter the correct items to complete the exercise. The entire tutorial takes about 20 min to complete.

### Intervention for the OT-MI Group

On top of the same OT received by the OT group, participants in the OT-MI group received MI via telephone. The interviewer made an appointment with the participants to perform a 15-min MI session over the phone after completing the baseline survey.

### Reminders for the OT and the OT-MI Groups

Reminders were sent via WeChat, WhatsApp, or emails to the participants of OT and OT-MI groups at 1, 2, 4, 6, and 8 months.

### Intervention for the Control Group

Participants in the control group received a link to access web-based health communication messages about the prevalence of some common mental health problems among MSM and the introduction of stress reduction exercises, which was totally unrelated to HPV or HPV vaccination. They did not receive any reminders.

### HPV Vaccination Uptake

If desired, then the interviewers would facilitate them to make an appointment to take up HPV vaccination in a collaborative private clinic at the market rate (HK $3800 [US $490] for 3 doses). No subsidy was provided. The authors did not limit participants’ choice to take up HPV vaccination at other places.

### Measures

#### Background Characteristics and Potential Confounders

Information collected included sociodemographic data (age, current marital status, educational level, current employment status, personal monthly income), sexual orientation, utilization of HIV or STI prevention services, history of HIV and other STIs, lifestyles (smoking and drinking), and sexual behaviors in the past 6 months. Queried sexual behaviors included anal intercourse with regular and nonregular sex partners, condomless anal intercourse (CAI) with men, multiple male sex partnerships, use of sexual potency drugs, and engagement in sexualized drug use. Regular male sex partners (RP) were defined as lovers and/or stable boyfriends, whereas nonregular partners (NRP) were defined as causal sex partners and/or male sex workers. In this study, sexualized drug use was defined as the use of any of the following psychoactive substances before or during sexual intercourse, including ketamine, methamphetamine, cocaine, cannabis, ecstasy, Dormicum/Halcion/Erimin 5/nonprescription hypnotic drugs, heroin, cough suppressant (not for curing cough), amyl nitrite (popper), GHB/GBL (γ-hydroxybutyrate), 5-methoxy-N, N-diisopropyltryptamine (Foxy), and mephedrone [40,41].

#### Primary Outcome

The primary outcome of the study was the validated completion of HPV vaccination within a 24-month follow-up period. To validate HPV vaccination uptake, the participants were requested to send the research team an image of their receipt hiding personal identification after they had received each dose of HPV vaccines. Supplemental information, including the location and cost of HPV vaccination uptake, was also collected.

#### Secondary Outcomes

Six scales assessed perceptions based on the HBM at baseline and 24 months. They were (1) the 4-item Perceived Susceptibility Scale; (2) the 2-item Perceived Severity Scale; (3) the 5-item Perceived Benefit Scale; (4) the 6-item Perceived Barrier Scale; (5) the 2-item Cue to Action Scale; and (6) the 3-item perceived self-efficacy scale. Cronbach α values for these 6 scales were acceptable (.72-.79). Single factors were identified for these scales in exploratory factor analysis, which explained 52.0%-71.0% of the total variance.

#### Postvaccination Experiences

Vaccinated participants were asked whether they had side effects of the vaccines (eg, pain, headache, fatigue, fever, nausea) and the severity of those side effects.

### Process Evaluation

At the end of the OT, participants were requested to answer 3 simple questions about some details of the video to verify their exposure. A similar approach was used by some other published studies [37]. The computer program recorded the starting and ending times of the OT. The fieldworkers recorded the starting and ending times of the MI for verification.

The process evaluation of health promotion was conducted at the third month. Participants in OT and OT-MI groups were asked (1) whether the content of the web-based video was clear; (2) whether the OT was attractive for you; and (3) whether the OT had increased their understanding of the benefit of HPV vaccination and willingness to take up HPV vaccination. Participants in the OT-MI group were asked some additional questions about their satisfaction with the MI session.

At 24 months, the participants were asked whether they had been exposed to any health communication messages promoting HPV vaccination during the project period. Those with such exposure were asked about some details, such as time, sources, contents, and the framing of such messages (ie, whether they supported or were against HPV vaccination).

### Sample Size Planning

Even without any intervention, about 30% intended to take up the HPV vaccine [27,28]. The meta-analysis showed that 43%-62% of those with a behavioral intention would translate it into action. In this study, the authors assumed that 12% of the participants in the OT group would complete HPV vaccination within the 24-month follow-up period. The authors expected that 2% of the participants in the control group would do so in the absence of any intervention. A sample size of 144 per group would allow us to detect a 10% (14/144) difference in the HPV vaccination uptake between the OT and control groups, and a 15% (22/144) difference between the OT-MI and OT groups (α=.0125; considering multiple comparisons, power of .8). With an expected loss-to-follow-up rate of 30% at 24 months, a sample size of approximately 208 per group is required (total n=624).

### Statistical Analysis

Intention-to-treat (ITT) analysis was used for the outcome analyses. Missing data were handled by the imputation strategy of last-observation-carried-forward, a standard method in the ITT analysis [42]. The chi-square test (for categorical variables) and one-way ANOVA (for continuous variables) were used to inspect the between-group balances of baseline characteristics. Logistic regression models (for primary outcome) and multivariable linear regression models (for secondary outcomes) were used to test the between-group differences in primary and secondary outcomes, after controlling for any background variables, showed *P*<.20 in between-group comparisons. Adjusted odds ratios (AORs) and adjusted standardized coefficients (β) were also obtained.

Baron and Kenny’s method was used to test the hypothesis that changes in the studied HBM constructs, which were used to design intervention that would mediate the between-group difference in the primary outcome [43]. Variables were constructed to assess changes in the HBM constructs (24 months-baseline). The mediation hypothesis was tested by first inspecting the association between changes in HBM constructs and randomization status. Logistic regression models were then fit using the validated uptake of 3 doses of HPV vaccination as the dependent variable and adjusted for significant baseline characteristics measured at baseline. Model 1 included randomization status as an independent variable, Model 2 included the changes in HBM constructs as the independent variables, and Model 3 included variables of both Models 1 and 2. If the association between randomization status and HPV vaccination uptake became statistically nonsignificant after adjusting for the changes in HBM constructs, the changes in HBM constructs were observed to fully mediate the association between randomization status and the dependent variable. Partial mediation occurred when the strength of association between randomization status and the dependent variable was reduced when remaining statistically significant changes in HBM constructs were controlled. SPSS version 16.0 was used, and *P* values <.05 were considered statistically significant.

## Results

### Descriptive Statistics

At baseline, the majority of the participants were single (510/624, 81.7%), full-time employed (492/624, 78.8%), had attended college or above (529/624, 84.8%), and identified themselves as gay (557/624, 89.3%). About one-third were aged between 18 and 26 years (202/624, 32.4%), and 50.2% (313/624) reported a monthly income of HK $20,000 (US $2580) or above. Regarding service utilization, 55.4% (346/624) and 48.1% (300/624) used HIV testing and other HIV or STI preventive services in the past 6 months. In the past 6 months, 80.4% (502/624) and 51.4% (321/324) had anal intercourse with RP and NRP, 35.3% (220/624) reported CAI with any men, 6.6% (41/624) used sexual potency drugs before or during anal intercourse, and 6.3% (39/624) reported sexualized drug use. The level of knowledge and perceptions related to HPV or HPV vaccination are shown in Table 1. Apart from anal intercourse with RP (*P*=.03) and the score of the perceived barrier scale (*P*=.03), no significant between-group difference was found (*P*=.24 to *P=*.96). Therefore, anal intercourse with RP and the score of the Perceived Barrier Scale were controlled in the subsequent analysis of primary and secondary outcomes.

**Table 1:** Characteristics of participants at baseline.

Characteristics			Control group (n=208)		OT^a^ group (n=208)	OT-MI^b^ group (n=208)		*P* value (comparing difference among control/OT/OT-MI groups)
**Sociodemographic data, n (%)**								
	**Age group (year)**						.57	
		18-26	65 (31.3)		73 (35.1)	64 (30.8)		
		27-36	101 (48.6)		91 (43.8)	91 (43.8)		
		37-45	42 (20.2)		44 (21.2)	53 (25.5)		
	**Current marital status**						.24	
		Currently single	171 (82.2)		162 (77.9)	177 (85.1)		
		Cohabited/married with a man	35 (16.8)		45 (40.5)	31 (14.9)		
		Cohabited/married with a woman	2 (1.0)		1 (0.5)	0 (0.0)		
	**Educational level**						.71	
		Secondary or below	29 (13.9)		31 (14.9)	35 (16.8)		
		University or above	179 (86.1)		177 (85.1)	173 (83.2)		
	**Current employment status**						.82	
		Full-time	166 (79.8)		165 (79.3)	161 (77.4)		
		Part-time/unemployed/retired/students	42 (20.2)		43 (20.7)	47 (22.6)		
	**Personal monthly income (HK $; US $)**						.38	
		<HK $10,000 (US $1290)	32 (15.4)	36 (17.3)		33 (15.9)		
		HK $10,000-$19,999 (US $1290-$2580)	74 (35.6)	71 (34.1)		62 (29.8)		
		HK $20,000-$39,999 (US $2580-$5161)	79 (38.0)	67 (32.2)		77 (37.0)		
		≥HK $40,000 (US $5161)	21 (10.1)	34 (16.3)		35 (16.8)		
		Refuse to disclose	2 (1.0)	0 (0.0)		1 (0.5)		
	**Sexual orientation**						.31	
		Gay	179 (86.1)	189 (90.9)		189 (90.9)		
		Bisexual	28 (13.5)	19 (9.1)		19 (9.1)		
		Heterosexual	1 (0.5)	0 (0.0)		0 (0.0)		
**HIV or STI^c^-related service use in the past 6 months, n (%)**								
	**HIV testing**						.55	
		No	93 (44.7)	98 (47.1)		87 (41.8)		
		Yes	115 (55.3)	110 (52.9)		121 (58.2)		
	**Other HIV or STI preventive services^d^**						.55	
		No	102 (49.0)	109 (52.4)		113 (54.3)		
		Yes	106 (51.0)	99 (47.6)		95 (45.7)		
**Sexual behaviors in the past six months** **, n (%)**								
	**Had anal intercourse with RP^e^**						.03	
		No	31 (14.9)	39 (18.8)		52 (25.0)		
		Yes	177 (85.1)	169 (81.3)		156 (75.0)		
	**Had anal intercourse with NRP^f^**						.93	
		No	103 (49.5)	101 (48.6)		99 (47.6)		
		Yes	105 (50.5)	107 (51.4)		109 (52.4)		
	**CAI^g^ with men**						.74	
		No	133 (63.9)	139 (66.8)		132 (63.5)		
		Yes	75 (36.1)	69 (33.2)		76 (36.5)		
	**Multiple male sex partnerships**						.88	
		No	90 (43.3)	92 (44.2)		95 (45.7)		
		Yes	118 (56.7)	116 (55.8)		113 (54.3)		
	**Sexualized drug use**						.56	
		No	193 (92.8)	198 (95.2)		194 (93.3)		
		Yes	15 (7.2)	10 (4.8)		14 (6.7)		
	**Use of sexual potency drugs**						.66	
		No	193 (92.8)	197 (94.7)		193 (92.8)		
		Yes	15 (7.2)	11 (5.3)		15 (7.2)		
**History of HIV or STI, n (%)**								
	**Self-reported HIV serostatus**						.96	
		Negative	183 (88.0)	185 (88.9)		184 (88.5)		
		Positive	11 (5.3)	7 (3.4)		8 (3.8)		
		Refuse to disclose	3 (1.4)	4 (1.9)		5 (2.4)		
		Had never tested for HIV antibody	11 (5.3)	12 (5.8)		11 (5.3)		
	**History of other STIs**						.61	
		No	170 (81.7)	170 (81.7)		163 (26.1)		
		Yes	38 (18.3)	38 (18.3)		45 (21.6)		
**Lifestyles, n (%)**								
	**Current smokers**						.88	
		No	165 (79.3)	162 (77.9)		166 (79.8)		
		Yes	43 (20.7)	46 (22.1)		42 (20.2)		
	**Drinking in the past year**						.32	
		No	39 (18.8)	28 (13.5)		36 (17.3)		
		Yes	169 (81.2)	180 (86.5)		172 (82.7)		
**Knowledge related to HPV^h^ or HPV vaccination, n (%)**								
	**Both males and females could be affected by HPV**						.06	
		Yes^i^	156 (75.0)	174 (83.7)		163 (78.4)		
		No	9 (4.3)	11 (5.3)		15 (7.2)		
		Do not know	43 (20.7)	23 (11.1)		30 (14.4)		
	**HPV infection could cause STI**						.63	
		Yes^i^	129 (62.0)	144 (69.2)		135 (64.9)		
		No	20 (9.6)	18 (8.7)		19 (9.1)		
		Do not know	59 (28.4)	46 (22.1)		54 (26.0)		
	**HPV infection could cause cancers among males**						.07	
		Yes^i^	89 (42.8)	114 (54.8)		99 (47.6)		
		No	34 (16.3)	37 (17.8)		37 (17.8)		
		Do not know	85 (40.9)	57 (27.4)		72 (34.6)		
	**HPV could be totally cured by available treatment**						.16	
		Yes	31 (14.9)	28 (13.5)		35 (16.8)		
		No^i^	110 (52.9)	132 (63.5)		112 (53.8)		
		Do not know	67 (32.2)	48 (23.1)		61 (29.1)		
	**Availability of effective HPV vaccination for males in Hong Kong**						.03	
		Yes^i^	113 (54.3)	131 (63.0)		110 (52.9)		
		No	18 (8.7)	28 (13.5)		26 (12.5)		
		Do not know	77 (37.0)	49 (23.6)		72 (34.6)		
	**Number of shots required to prevent HPV infection in males**						.31	
		3	56 (26.9)	69 (33.2)		68 (32.7)		
		Other answers/Do not know	152 (73.1)	139 (66.8)		140 (67.3)		
	**Number of correct responses**						.26	
		0	33 (15.9)	19 (9.1)		27 (13.0)		
		1-2	45 (21.6)	40 (19.2)		46 (22.1)		
		3-4	100 (48.1)	104 (50.0)		95 (47.9)		
		5-6	30 (14.4)	45 (21.6)		40 (19.2)		
**Perceptions related to HPV or HPV vaccination based on the HBM^j^**								
	**Perceived susceptibility to HPV (high/very high)**							
		Perceived risk of contracting HPV in lifetime, n (%)	42 (20.2)	52 (25.0)		49 (23.6)		.49
		Perceived risk of contracting genital warts in lifetime, n (%)	39 (18.8)	51 (24.5)		47 (22.6)		.35
		Perceived risk of having penile or anal cancers in lifetime, n (%)	20 (9.6)	25 (12.0)		21 (10.1)		.70
		Perceived HPV infection rate among MSM^k^ in Hong Kong, n (%)	63 (30.3)	53 (25.5)		55 (26.4)		.51
		Perceived susceptibility scale, mean (SD)^i^	10.8 (3.0)	10.9 (3.3)		10.4 (3.5)		.38
	**Perceived severity of HPV-related diseases (high/very high)**							
		Harms of genital warts on physical health, n (%)	119 (57.2)	119 (57.2)		128 (61.5)		.59
		Harms of penile or anal cancers on physical health, n (%)	135 (64.9)	153 (73.6)		137 (65.9)		.12
		Perceived severity scale, mean (SD)^l^	7.6 (1.9)	7.7 (1.7)		7.5 (1.8)		.59
	**Perceived benefit of HPV vaccination (agree/strongly agree)**							
		HPV vaccination is highly effective in preventing HPV infection, n (%)	143 (68.8)	166 (79.8)		147 (70.7)		.03
		HPV vaccination is highly effective in preventing genital warts, n (%)	142 (68.3)	151 (72.6)		138 (66.3)		.37
		HPV vaccination is highly effective in preventing penile/anal cancers, n (%)	129 (62.0)	138 (66.3)		123 (59.1)		.31
		HPV vaccination can protect you for a long time, n (%)	104 (50.0)	113 (54.3)		105 (50.5)		.63
		You will feel at ease after receiving HPV vaccination, n (%)	142 (68.3)	127 (61.1)		139 (66.8)		.26
		Perceived benefit scale, mean (SD)^m^	18.9 (3.1)	18.9 (2.9)		18.6 (2.9)		.58
	**Perceived barriers of receiving HPV vaccination (agree/strongly agree)**							
		It is not worthy spending HK $2000-$3000 (US $257.97-$386.96) to receive HPV vaccination, n (%)	64 (30.8)	44 (21.1)		38 (18.3)		.01
		The procedures to receive HPV vaccination is troublesome, n (%)	33 (15.9)	30 (13.3)		17 (8.2)		.05
		You would have severe side effects after receiving HPV vaccination, n (%)	20 (9.6)	15 (7.2)		17 (8.2)		.67
		You feel embarrassed to receive HPV vaccination, n (%)	25 (12.0)	20 (9.6)		18 (8.7)		.50
		Others would think you are having high-risk behaviors if you receive HPV vaccination, n (%)	33 (15.9)	25 (12.0)		25 (12.0)		.41
		You would be stigmatized by service providers when you receive HPV vaccination, n (%)	18 (8.7)	15 (7.2)		13 (6.3)		.64
		Perceived barrier scale, mean (SD)^n^	13.5 (5.2)	12.6 (4.0)		12.4 (4.2)		.03
	**Perceived cue to action related to HPV vaccination (agree/strongly agree)**							
		Medical professionals would suggest you to receive HPV vaccination, n (%)	3 (1.4)	6 (2.9)		7 (3.4)		.43
		MSM peers would suggest you to receive HPV vaccination, n (%)	8 (3.8)	15 (7.2)		5 (2.4)		.06
		Cue to action scale, mean (SD)^o^	2.7 (1.3)	2.9 (1.5)		2.8 (1.4)		.56
	**Perceived self-efficacy related to HPV vaccination (agree/strongly agree)**							
		Whether to receive HPV vaccination is completely under your control, n (%)	172 (82.7)	169 (81.3)		183 (88.0)		.14
		You are confident to receive HPV vaccination in the next year if you want, n (%)	142 (68.3)	139 (66.8)		147 (70.7)		.70
		Receiving HPV vaccination in the next year is easy for you if you want, n (%)	154 (74.0)	145 (69.7)		153 (73.6)		.56
		Perceived self-efficacy scale, mean (SD)^p^	12.6 (2.4)	12.4 (2.6)		12.6 (2.4)		.61

^a^OT: online tutorial.

^b^MI: motivational interviewing.

^c^STI: sexually transmitted infection.

^d^Including receiving condoms, peer education, leaflets of HIV-related information, and seminars or workshops related to HIV.

^e^RP: regular male sex partners.

^f^NRP: nonregular partners.

^g^CAI: condomless anal intercourse.

^h^HPV: human papillomavirus.

^i^Perceived susceptibility scale, 3 items, Cronbach α=.79, one factor was identified by exploratory factor analysis, which explained 61.5% of the total variance.

^j^HBM: health belief model.

^k^MSM: men who have sex with men.

^l^Perceived severity scale, 2 items, Cronbach α=.64.

^m^Perceived benefit scale, 5 items, Cronbach α=.75, one factor was identified by exploratory factor analysis, explaining 52.0% of the total variance.

^n^Perceived barrier scale, 6 items, Cronbach α=.86, one factor was identified by exploratory factor analysis, explaining 71.0% of the total variance.

^o^Cue to action scale, 2 items, Cronbach α=.62.

^p^Perceived self-efficacy scale: 3 items, Cronbach α=.78, one factor was identified by exploratory factor analysis, explaining 69.2% of total variance.

The loss-to-follow-up rates in the control, OT, and OT-MI groups at 24 months were 24.5% (51/208), 31.3% (65/208), and 23.6% (49/208), respectively. Significant differences in the current marital status, educational level, anal intercourse with NRP, CAI with men, multiple male sex partnerships, sexualized drug use, and the use of sexual potency drugs were found in at least one group while comparing those who were followed up and those who were lost to follow-up at 24 months (Multimedia Appendix 1).

### Between-Group Difference in Primary Outcome

All 75 participants who self-reported having had completed HPV vaccination at 24 months were able to provide receipts for verification. Participants in the OT-MI group reported significantly higher HPV vaccination completion rates at 24 months than that by the control group (36/208, 17.3% vs 15/208, 7.2%; AOR 1.57, 95% CI 1.14-2.17; *P*=.006). However, the difference in HPV vaccination completion between the OT and the control groups (24/208, 11.5% vs 15/208, 7.2%; AOR 1.61, 95% CI 0.82-3.18; *P*=.17), or between OT-MI and OT groups (36/208, 17.3% vs 24/208, 11.5%, AOR 1.55, 95% CI 0.89-2.72; *P*=.13), was not statistically significant.

The location for receiving HPV vaccination included the collaborative private clinic (54/75, 72%), other private clinics in Hong Kong (19/75, 25%), university health care center (1/75, 1%), and clinics in Australia (1/75, 1%). Except for one participant who received free HPV vaccination in Australia, other participants self-paid HK $1600 to HK $9000 (US $206-US $1161; median: HK $3800 or US $490) to receive the vaccination. The majority of the vaccinated participants completed the entire course within 12 months (55/75; 73%), whereas 20 (20/75, 27%) completed it within 24 months.

### Between-Group and Within-Group Differences in Secondary Outcomes

Compared with the participants in the control group, participants in the OT-MI (adjusted β: –.21; *P*<.001) and OT (adjusted β: –.10; *P*=.02) groups reported lower perceived barriers to taking up HPV vaccination at 24 months. The comparison of 24-month and baseline data revealed statistical increase in perceived severity (in all 3 groups, *P*<.001) and perceived barriers (in the control group and the OT group, *P*<.001 and *P*=.001, respectively). A statistically significant decrease in perceived benefit was found in the control group (*P*=.04), whereas a decrease in perceived self-efficacy was found in both the control (*P*=.05) and the OT-MI groups (*P*=.002; Table 2 and Multimedia Appendix 1).

**Table 2:** Between-group comparisons of secondary outcomes.

Secondary outcomes (perceptions based on the HBM^a^)		OT^b^ group vs control group		OT-MI^c^ group vs control group		OT-MI group vs OT group		
		Adjusted β^d^	*P* value	Adjusted β	*P* value		Adjusted β	*P* value
**Perceived susceptibility scale**								
	Baseline	.01	.91	–.07	.19		–.06	.22
	24 months	–.01	.88	.05	.26		.06	.23
	24 months-Baseline	–.01	.79	.12	.02		.11	.02
**Perceived severity scale**								
	Baseline	.04	.37	.01	.82		–.04	.38
	24 months	.01	.80	.03	.62		.02	.68
	24 months-Baseline	–.03	.52	.02	.75		.06	.24
**Perceived benefit scale**								
	Baseline	–.02	.70	–.06	.20		–.05	.30
	24 months	–.001	.98	.03	.53		.02	.69
	24 months-Baseline	.02	.75	.09	.09		.07	.19
**Perceived barrier scale**								
	Baseline	–.10	.05	–.12	.02		–.03	.60
	24 months	–.11	.01	–.21	<.001		–.10	.02
	24 months-Baseline	–.12	.01	–.21	<.001		–.11	.02
**Cue to action scale**								
	Baseline	.06	.26	.02	.68		–.04	.48
	24 months	–.004	.93	.01	.78		.01	.87
	24 months-Baseline	–.05	.34	–.003	.95		.03	.49
**Perceived self-efficacy scale**								
	Baseline	–.08	.10	–.05	.29		.03	.55
	24 months	–.06	.22	–.09	.08		–.04	.45
	24 months-Baseline	.02	.66	–.03	.53		–.06	.23

^a^HBM: health belief model.

^b^OT: online tutorial.

^c^MI: motivational interviewing.

^d^Standardized coefficients adjusted for anal intercourse with regular male sex partners and score of Perceived Barrier Scale measured at baseline.

### Testing the Mediation Hypotheses

Adjusted for potential confounders assessed at baseline, the within-group changes in the scores of the Perceived Susceptibility Scale and Perceived Barrier Scale were significantly associated with the intervention status (the OT-MI group vs the control group, *P*=.02 and *P*<.001, respectively) and HPV vaccination completion (*P*<.001, Table 3).

The association between the intervention status (OT-MI vs control) and HPV vaccination completion became statistically nonsignificant (AOR 1.42, 95% CI 0.99-2.03; *P*=.06) after controlling for the change in perceived susceptibility, whereas the change in perceived susceptibility remained strongly associated with the dependent variable in the model (AOR 1.23, 95% CI 1.12-1.35; *P*<.001). The results suggested that changes in perceived susceptibility fully mediated the intervention effect. The association between intervention status and HPV vaccination completion was weakened (from *P*<.001 to *P*=.05) when changes in perceived barriers were controlled in the model, with the change in perceived barrier remaining statistically significant in the model (*P*<.001). A partial mediation effect was observed (Table 3).

**Table 3:** Test for the independent effect of changes in Health Belief Model scale scores (24 months vs baseline) on the association between intervention status (online tutorial- motivational interviewing group vs control group) and human papillomavirus vaccination completion during the follow-up period (n=416).

Model and variables		Baseline	SE	AOR^a,b^ (95% CI)	*P* value
**1**					
	Intervention status (OT^c^-MI^d^ group vs control group)	0.47	0.17	1.61 (1.14-2.26)	.006
**2A**					
	Change in score of the perceived susceptibility scale	0.22	0.05	1.25 (1.14-1.38)	<.001
**3A**					
	Intervention status (OT-MI group vs control group)	0.35	0.18	1.42 (0.99-2.03)	.06
	Change in score of the perceived susceptibility scale	0.21	0.05	1.23 (1.12-1.35)	<.001
**2B**					
	Change in score of the perceived barrier scale	–0.18	0.04	0.84 (0.77-0.91)	<.001
**3B**					
	Intervention status (OT-MI group vs control group)	0.37	0.18	1.44 (1.01-2.06)	.05
	Change in score of the perceived barrier scale	–0.16	0.04	0.85 (0.79-0.92)	<.001

^a^AOR: adjusted odds ratios

^b^Odds ratios adjusted for potential confounders measured at baseline (sociodemographic data, HIV and sexually transmitted disease [STI]–related service utilization, history of HIV and STI, sexual behaviors, lifestyles, and knowledge related to human papillomavirus [HPV]/HPV vaccination).

^c^OT: online tutorial.

^d^MI: motivational interviewing.

### Postvaccination Experiences

The majority of the vaccinated participants reported no side effects (65/75, 87%). The reported side effects included pain at the injection site (4/75, 5%), fever (3/75, 4%), numbness in arms (2/75, 3%), nausea (2/75, 3%), fatigue (1/75, 1%), and headache (1/75, 1%). Most of these side effects were mild (8/10; 80%).

### Process Evaluation of Web-based Intervention Promoting HPV Vaccination

As recorded by the computer program, all participants in the OT and the OT-MI groups completed the OT. The time spent on the tutorial ranged from 15 to 28 min. The duration of MI ranged from 11 to 19 min.

Among those in the OT and OT-MI groups who participated in the process evaluation at 3 months, 84.1% (281/334) and 46.1% (154/334) believed that the content of the web-based health promotion video was clear and attractive. Moreover, 77.2% (258/334) and 47.0% (157/334) indicated that the OT has increased their understanding of HPV vaccination and willingness to take up HPV vaccination. Among those in the OT-MI group, 86.8% (159/182) were satisfied with the MI session, and 60.9% (111/182) believed that the MI session reduced their barriers toward getting HPV vaccination.

Among participants who completed the survey at 24 months 6.5% (30/459) had been exposed to other health communication messages that supported HPV vaccination during the project period, whereas 3.1% (14/459) had been exposed to other health communication messages that were against HPV vaccination during the same period. The most common resource of these health communication messages was pamphlets, followed by communication with peers and web-based communication.

## Discussion

### Principal Findings

Compared with the control group, the OT plus MI over the phone brought a significant increase in HPV vaccination completion among MSM over a 24-month follow-up period (36/208, 17.3% vs 15/208, 7.2%; *P*=.006). However, the hypothesis that using OT alone would show superior results as compared with the control group (24/208, 11.5% vs 15/208, 7.2%; *P*=.17) and adding MI to the OT would be more efficacious (36/208, 17.3% vs 24/208, 11.5%; *P*=.13) were not supported by the results.

The net increase in HPV vaccination uptake observed in this study (OT-MI group vs control group, 10.1%) was slightly higher than that of the other web-based interventions targeting MSM in the United States (8%) [29]. Unlike in the United States, where the cost of HPV vaccination can be fully covered by multiple sources of private and public financing [44], users self-paid for HPV vaccination at the market price without any subsidy in this study. Given the high HPV-related disease burden among Chinese MSM, instead of waiting for the free HPV vaccination program to become available, there is an urgent need for promoting self-paid HPV vaccination among MSM in Hong Kong. For the first time, health workers in Hong Kong were provided with an evidence-based intervention for promoting HPV vaccination among MSM. The authors’ intervention had some strengths. First, no additional funding is required to pay for HPV vaccination in this intervention. Second, the OT required minimum resources for maintenance. With simple training, health workers can easily perform brief MI. Therefore, governmental and NGOs can integrate this intervention into their existing HIV or STI prevention services and scale it up with little extra cost. The research team successfully embedded the intervention into HIV testing and counseling services run by a local NGO [45]. MSM using the service completes the OT on a tablet while they are waiting for their testing results. The NGO also enhanced posttest counseling to include brief MI promoting HPV vaccination for the users.

Given that the HPV vaccination uptake in the control group was higher than expected, and the difference between OT and control groups was smaller than expected, we believe that the nonsignificant findings may be attributed to an inadequate sample size and a limited statistical power. The *P* values for the comparison between the OT and control groups, and between the OT-MI and OT groups were close to .10. Future studies with larger sample sizes should be conducted. Therefore, the authors recommend OT together with MI for HPV vaccination promotion among MSM in Hong Kong until further evidence is generated.

The intervention also caused between-group differences in perceptions related to the HBM that were used to develop the intervention. The mediation analysis results explained some plausible mechanisms that might cause the observed behavioral change. The behavioral change may be caused by an increase in perceived susceptibility to HPV or HPV-related diseases. This finding was consistent with studies that demonstrated a strong association between risk perception and behavioral change in different health topics [46,47]. In the absence of intervention, perceived barriers to taking up HPV vaccination increased over time in the control group. Receiving OTs and brief MI over the phone slowed down the increase in perceived barriers. The between-group (OT-MI vs control) difference in changes of perceived barriers partially mediated the intervention effects. This study extended the applicability of HBM.

This study had the strength of its RCT design, long follow-up duration, well-validated primary outcome, and was based on theory and supported by the results of formative studies. The intervention was well received based on the positive process evaluation results. However, this study has some limitations. First, the intervention was limited to MSM who had internet access, but the majority of MSM in Hong Kong is expected to have access to the internet as the smartphone ownership is above 96% [48]. It is justifiable that the penetration of smartphones has been increasing sharply in many countries. Second, probability sampling was not feasible for this study in the absence of a sampling frame. Like most RCTs, the participants were recruited by convenient sampling, and selection bias might exist. The RCT design ensured a good internal validity. However, caution should be exercised while generalizing the results to other Chinese cities. Third, attrition bias might exist. The dropouts in OT-MI group had a higher prevalence of sexual risk behaviors than nondropouts, and they may have a higher motivation to take up HPV vaccination. The HPV uptake rate in the OT-MI group might be underestimated. Moreover, we did not collect information about MSM who refused to participate in the study. Furthermore, evaluating the MI sessions by audiotaping was the gold standard to assess fidelity. However, the authors were not able to do so, as the participants were MSM, and the studied questions covered sensitive topics such as their HIV and STI status and sexual behaviors.

### Conclusions

The RCT findings showed that the theory-based OT, together with brief MI over the phone, was effective in increasing the completion of 3 required doses of HPV vaccination among MSM in Hong Kong, China. At present, local NGOs have integrated interventions in their HIV testing and counseling services for MSM. Local and international dissemination and implementation research are greatly warranted.

## Appendix

Multimedia Appendix 1 Comparing baseline characteristics between participants being followed up and those who were lost-to-follow-up at Month 24 and descriptive statistics of primary and secondary outcomes at baseline and Month 24 follow-up.

## Abbreviations

AOR: Adjusted odds ratios
CAI: condomless anal intercourse
CDC: Centers for Disease Control and Prevention
HBM: Health Belief Model
HPV: human papillomavirus
ITT: intention-to-treat
mHealth: mobile health
MI: motivational interviewing
MSM: men who have sex with men
NGO: nongovernmental organization
NRP: nonregular partners
OT: online tutorial
RCT: randomized controlled trial
RP: Regular male sex partners
STI: sexually transmitted infection
