# Supplementary material for: Two Web-Based and Theory-Based Interventions With and Without Brief Motivational Interviewing in the Promotion of Human Papillomavirus Vaccination Among Chinese Men Who Have Sex With Men: Randomized Controlled Trial
Source: J Med Internet Res. 2021 Feb 2;23(2):e21465. doi: 10.2196/21465 (PMC7886619; doi:10.2196/21465)
Supplement: Multimedia Appendix 1 [file jmir_v23i2e21465_app1.docx]

Table S1 Comparing baseline characteristics between participants being followed up and those who were lost-to-follow-up at Month 24

|  | Control group (n=208) | | | HC group (n=208) | | | HC-MI group (n=208) | | |
| --- | --- | --- | --- | --- | --- | --- | --- | --- | --- |
|  | Being followed-up at Month 24  (n=157) | Loss to follow-up  (n=51) |  | Being followed-up at Month 24  (n=143) | Loss to follow-up  (n=65) |  | Being followed-up at Month 24  (n=159) | Loss to follow-up  (n=49) |  |
|  | % | % | P value | % | % | P value | % | % | P value |
| **Socio-demographics** |  |  |  |  |  |  |  |  |  |
| Age group (year) |  |  |  |  |  |  |  |  |  |
| 18-26 | 33.1 | 25.5 | .44 | 32.2 | 41.5 | .34 | 29.6 | 34.7 | .53 |
| 27-36 | 48.4 | 49.0 |  | 46.9 | 36.9 |  | 45.9 | 36.7 |  |
| 37-45 | 18.5 | 25.5 |  | 21.0 | 21.5 |  | 24.5 | 28.6 |  |
| Current marital status |  |  |  |  |  |  |  |  |  |
| Currently single | 82.2 | 82.4 | .04 | 76.9 | 80.0 | .73 | 85.5 | 83.7 | .75 |
| Cohabited/married with a man | 17.8 | 13.7 |  | 22.4 | 20.0 |  | 14.5 | 16.3 |  |
| Cohabited/married with a woman | 0.0 | 3.9 |  | 0.7 | 0.0 |  | 0.0 | 0.0 |  |
| Educational level |  |  |  |  |  |  |  |  |  |
| Secondary or below | 12.1 | 19.6 | .18 | 15.4 | 13.8 | .77 | 13.2 | 28.6 | .01 |
| University or above | 87.9 | 80.4 |  | 84.6 | 86.2 |  | 86.8 | 71.4 |  |
| Current employment status |  |  |  |  |  |  |  |  |  |
| Full-time | 79.6 | 80.4 | .91 | 79.7 | 78.5 | .84 | 75.5 | 83.7 | .23 |
| Part-time/ unemployed/retired/students | 20.4 | 19.6 |  | 20.3 | 21.5 |  | 24.5 | 16.3 |  |
| Personal monthly income (HK$) |  |  |  |  |  |  |  |  |  |
| <10,000 | 17.2 | 9.8 | .07 | 18.2 | 15.4 | .83 | 17.6 | 10.2 | .08 |
| 10,000-19,999 | 36.9 | 31.4 |  | 33.6 | 35.4 |  | 25.2 | 44.9 |  |
| 20,000-39,999 | 36.3 | 43.1 |  | 30.8 | 35.4 |  | 40.3 | 26.5 |  |
| ≥40,000 | 9.6 | 11.8 |  | 17.5 | 13.8 |  | 16.4 | 18.4 |  |
| Refuse to disclose | 0.0 | 3.9 |  | 0.0 | 0.0 |  | 0.6 | 0.0 |  |
| Sexual orientation |  |  |  |  |  |  |  |  |  |
| Gay | 86.0 | 86.3 | .20 | 90.9 | 90.8 | .97 | 91.2 | 89.8 | .77 |
| Bisexual | 14.0 | 11.8 |  | 9.1 | 9.2 |  | 8.8 | 10.2 |  |
| Heterosexual | 0.0 | 2.0 |  | 0.0 | 0.0 |  | 0.0 | 0.0 |  |
| **HIV/sexually transmitted diseases (STI)-related service utilization in the past six months** |  |  |  |  |  |  |  |  |  |
| HIV testing |  |  |  |  |  |  |  |  |  |
| No | 43.9 | 47.1 | .70 | 47.6 | 46.2 | .85 | 42.8 | 38.8 | .62 |
| Yes | 56.1 | 52.9 |  | 52.4 | 53.8 |  | 57.2 | 61.2 |  |
| Other HIV/STI preventive services ^a^ |  |  |  |  |  |  |  |  |  |
| No | 47.8 | 52.9 | .52 | 51.7 | 53.8 | .78 | 54.1 | 55.1 | .90 |
| Yes | 52.2 | 47.1 |  | 48.3 | 46.2 |  | 45.9 | 44.9 |  |
| **Sexual behaviors in the past six months** |  |  |  |  |  |  |  |  |  |
| Had had anal intercourse with regular male sex partners (RP) |  |  |  |  |  |  |  |  |  |
| No | 13.4 | 19.6 | .28 | 19.6 | 16.9 | .65 | 25.8 | 22.4 | .64 |
| Yes | 76.8 | 80.4 |  | 80.4 | 83.1 |  | 74.2 | 77.6 |  |
| Had had anal intercourse with non-regular male sex partners (NRP) |  |  |  |  |  |  |  |  |  |
| No | 49.0 | 51.0 | .81 | 49.7 | 46.2 | .64 | 51.6 | 34.7 | .04 |
| Yes | 51.0 | 49.0 |  | 50.3 | 53.8 |  | 48.4 | 65.3 |  |
| Condomless anal intercourse (CAI) with men |  |  |  |  |  |  |  |  |  |
| No | 59.5 | 76.5 | .03 | 65.7 | 69.2 | .62 | 65.4 | 57.1 | .29 |
| Yes | 40.1 | 23.5 |  | 34.3 | 30.8 |  | 34.6 | 42.9 |  |
| Multiple male sex partnerships |  |  |  |  |  |  |  |  |  |
| No | 43.9 | 41.2 | .73 | 47.6 | 36.9 | .15 | 50.9 | 28.6 | .01 |
| Yes | 56.1 | 58.8 |  | 52.4 | 63.1 |  | 49.1 | 71.4 |  |
| Sexualized drug use |  |  |  |  |  |  |  |  |  |
| No | 93.6 | 90.2 | .41 | 95.8 | 93.8 | .54 | 97.5 | 79.6 | <.001 |
| Yes | 6.4 | 9.8 |  | 4.2 | 6.2 |  | 2.5 | 20.4 |  |
| Use of sexual potency drugs |  |  |  |  |  |  |  |  |  |
| No | 94.9 | 86.3 | .04 | 96.5 | 90.8 | .09 | 93.7 | 89.8 | .35 |
| Yes | 5.1 | 13.7 |  | 3.5 | 9.2 |  | 6.3 | 10.2 |  |
| **History of HIV/STI** |  |  |  |  |  |  |  |  |  |
| Self-reported HIV sero-status |  |  |  |  |  |  |  |  |  |
| Negative | 87.3 | 90.2 | .73 | 88.1 | 90.8 | .47 | 88.1 | 89.8 | .53 |
| Positive | 5.1 | 5.9 |  | 2.8 | 4.6 |  | 3.1 | 6.1 |  |
| Refuse to disclose | 1.9 | 0.0 |  | 2.8 | 0.0 |  | 2.5 | 2.0 |  |
| Had never tested for HIV antibody | 5.7 | 3.9 |  | 6.3 | 4.6 |  | 6.3 | 2.0 |  |
| History of other STI |  |  |  |  |  |  |  |  |  |
| No | 82.2 | 80.4 | .78 | 81.8 | 81.5 | .96 | 78.6 | 77.6 | .87 |
| Yes | 17.8 | 19.6 |  | 18.2 | 18.5 |  | 21.4 | 22.4 |  |
| **Lifestyles** |  |  |  |  |  |  |  |  |  |
| Current smokers |  |  |  |  |  |  |  |  |  |
| No | 79.0 | 80.4 | .83 | 79.0 | 75.4 | .56 | 84.3 | 65.3 | .004 |
| Yes | 21.0 | 19.6 |  | 21.0 | 24.6 |  | 15.7 | 34.7 |  |
| Drinking in the past year |  |  |  |  |  |  |  |  |  |
| No | 20.4 | 13.7 | .29 | 11.2 | 18.5 | .15 | 15.1 | 24.5 | .13 |
| Yes | 79.6 | 86.3 |  | 88.8 | 81.5 |  | 84.9 | 75.5 |  |
| **Knowledge related to HPV/HPV vaccination** |  |  |  |  |  |  |  |  |  |
| Number of correct responses |  |  |  |  |  |  |  |  |  |
| 0 | 14.0 | 21.6 | .63 | 9.8 | 7.7 | .09 | 12.6 | 14.3 | .57 |
| 1-2 | 21.7 | 21.6 |  | 29.2 | 14.7 |  | 21.4 | 24.5 |  |
| 3-4 | 49.7 | 43.1 |  | 41.5 | 53.8 |  | 44.7 | 49.0 |  |
| 5-6 | 14.6 | 13.7 |  | 21.7 | 21.5 |  | 21.4 | 12.2 |  |
| **Perceptions related to HPV/HPV vaccination based on the Health Belief Model (HBM)** |  |  |  |  |  |  |  |  |  |
| Perceived Susceptibility Scale (mean/SD) | 10.8 (3.1) | 10.7 (2.8) | .78 | 10.9 (3.4) | 10.8 (3.3) | .88 | 10.3 (3.5) | 11.0 (3.4) | .24 |
| Perceived Severity Scale  (mean/SD) | 7.6 (1.8) | 7.3 (2.0) | .32 | 7.7 (1.6) | 7.6 (1.9) | .64 | 7.5 (1.8) | 7.5 (1.8) | .88 |
| Perceived Benefit Scale  (mean/SD) | 19.1 (3.2) | 18.4 (2.7) | .18 | 18.9 (2.6) | 18.8 (3.4) | .66 | 18.7 (2.9) | 18.3 (2.6) | .41 |
| Perceived Barrier Scale  (mean/SD) | 13.9 (5.2) | 12.3 (5.0) | .06 | 12.7 (3.9) | 12.4 (4.4) | .62 | 12.4 (4.2) | 12.4 (4.2) | .93 |
| Cue to Action Scale  (mean/SD) | 2.8 (1.3) | 2.6 (1.2) | .34 | 3.0 (1.6) | 2.6 (1.1) | .04 | 2.67 (1.2) | 3.1 (1.8) | .17 |
| Perceived Self-efficacy Scale  (mean/SD) | 12.7 (2.4) | 12.5 (2.3) | .53 | 12.4 (2.4) | 12.5 (2.8) | .68 | 12.6 (2.5) | 12.8 (2.2) | .62 |

Table S2 Descriptive statistics of primary and secondary outcomes at baseline and 24 months follow-up

|  | Control group | HC group | HC-MI group |
| --- | --- | --- | --- |
|  | n (%) | n (%) | n (%) |
| **Primary outcome** |  |  |  |
| HPV vaccination completion (receipt of three required doses) within 24-month follow-up period | 15/208  (7.2%) | 24/208  (11.5%) | 36/208  (17.3%) |
|  | Mean (SD) | Mean (SD) | Mean (SD) |
| **Secondary outcomes**  Perceptions based on the HBM |  |  |  |
| Perceived Susceptibility Scale |  |  |  |
| Baseline | 10.8 (3.0) | 10.9 (3.3) | 10.4 (3.5) |
| 24 months | 10.6 (3.1) | 10.5 (3.5) | 11.0 (3.4) |
| 24 months–Baseline | -0.2 (3.1) | -0.3 (3.5) | 0.5 (3.8) |
| *P value ^a^* | .29 | .19 | .05 |
| Perceived Severity Scale |  |  |  |
| Baseline | 7.6 (1.9) | 7.7 (1.7) | 7.5 (1.8) |
| 24 months | 8.7 (1.7) | 8.8 (1.6) | 8.8 (1.5) |
| 24 months–Baseline | 1.2 (1.9) | 1.1 (1.7) | 1.3 (1.9) |
| *P value ^a^* | <.001 | <.001 | <.001 |
| Perceived Benefit Scale |  |  |  |
| Baseline | 18.9 (3.1) | 18.9 (2.9) | 18.6 (2.9) |
| 24 months | 18.4 (3.0) | 18.5 (3.4) | 18.7 (3.0) |
| 24 months–Baseline | -0.5 (3.4) | -0.3 (3.2) | 0.1 (3.3) |
| *P value ^a^* | .04 | .12 | .82 |
| Perceived Barrier Scale |  |  |  |
| Baseline | 13.5 (5.2) | 12.6 (4.0) | 12.4 (4.2) |
| 24 months | 15.0 (4.6) | 13.4 (4.3) | 12.5 (4.1) |
| 24 months–Baseline | 1.4 (4.8) | 0.8 (3.5) | 0.1 (4.1) |
| *P value ^a^* | <.001 | .001 | .79 |
| Cue to Action Scale |  |  |  |
| Baseline | 2.7 (1.3) | 2.9 (1.5) | 2.8 (1.4) |
| 24 months | 2.9 (1.6) | 2.9 (1.5) | 2.9 (1.5) |
| 24 months–Baseline | 0.2 (1.8) | 0.03 (1.8) | 0.1 (1.9) |
| *P value ^a^* | .14 | .79 | .27 |
| Perceived Self-efficacy Scale |  |  |  |
| Baseline | 12.6 (2.4) | 12.4 (2.6) | 12.6 (2.4) |
| 24 months | 12.3 (2.1) | 12.1 (2.5) | 12.0 (2.5) |
| 24 months–Baseline | -0.3 (2.5) | -0.3 (2.6) | -0.6 (2.8) |
| *P value ^a^* | .05 | .12 | .002 |

*^a^ Comparing 24 months vs baseline* *by using paired sample t tests*
